# Antioxidant, Phytochemical, and Pharmacological Properties of Algerian *Mentha aquatica* Extracts

**DOI:** 10.3390/antiox13121512

**Published:** 2024-12-11

**Authors:** Radhia Aitfella Lahlou, Ana Carolina Gonçalves, Mustapha Bounechada, Ana R. Nunes, Pedro Soeiro, Gilberto Alves, Diego A. Moreno, Cristina Garcia-Viguera, Cesar Raposo, Samuel Silvestre, Jesus M. Rodilla, Maria Isabel Ismael, Luís R. Silva

**Affiliations:** 1 Chemistry Department, University of Beira Interior, 6201-001 Covilhã, Portugal; sms@ubi.pt (S.S.); rodilla@ubi.pt (J.M.R.);; 2 Fiber Materials and Environmental Technologies (FibEnTech), University of Beira Interior, 6201-001 Covilhã, Portugal; 3 RISE-Health, Faculdade de Ciências da Saúde, Universidade da Beira Interior, Av. Infante D. Henrique, 6200-506 Covilhã, Portugal; anacarolinagoncalves@sapo.pt (A.C.G.); pedroafsoeiro@gmail.com (P.S.); gilberto@fcsaude.ubi.pt (G.A.); luisfarmacognosia@gmail.com (L.R.S.); 4 SPRINT Sport Physical Activity and Health Research & Innovation Center, Instituto Politécnico da Guarda, 6300-559 Guarda, Portugal; 5 University Ferhat Abbes Sétif1, Faculty of Natural Sciences and Life, 19000, Algeria; bounechadam@yahoo.fr; 6 Laboratorio de Fitoquímica y Alimentos Saludables” (LabFAS), CSIC, CEBAS, Campus Universitario de Espinardo-25, E-30100 Murcia, Spain; dmoreno@cebas.csic.es (D.A.M.); cgviguera@cebas.csic.es (C.G.-V.); 7 Mass Spectrometry Service, University of Salamanca, 37007 Salamanca, Spain; 8 Centro de Química Estrutural, Institute of Molecular Sciences, Faculdade de Ciências, Universidade de Lisboa, Campo Grande, 1749-016 Lisboa, Portugal

**Keywords:** *Mentha aquatica*, phenolic compounds, antioxidant, antidiabetic, lipid peroxidation, hemoglobin oxidation, cytotoxicity

## Abstract

Water mint (*Mentha aquatica*) is used in many formulations worldwide as a functional food and natural remedy to treat gastrointestinal disorders, lung diseases, and certain mental disorders such as epilepsy and depression. This study assessed the bioactivity of its infusion extract (INF) and hydroethanolic extract (HE) to highlight its health benefits. These extracts were analyzed for their chemical composition by HPLC-DAD-ESI-MSn, their antioxidant and antidiabetic properties, and their capacities to protect human erythrocytes against induced hemoglobin oxidation and lipid peroxidation. The effect on normal human dermal fibroblast (NHDF) cells and on the N27 rat dopaminergic neuron cell line was also assessed. The chromatographic analysis identified 57 compounds belonging to hydroxycinnamic acids, flavanones, flavone, and isoflavonoids. In respect to the biological potential, the *Mentha aquatica* extracts revealed a notable capacity for 2,2-diphenyl-1-picrylhydrazyl, nitric oxide, and superoxide radicals, as well as for the inhibition of *α*-glucosidase action and the protection of human erythrocytes against oxidative damage. Quantification revealed noteworthy phenolic content in both extracts. Additionally, the extracts demonstrated less cytotoxic effects regarding the NHDF and N27 cell lines. Overall, *Mentha aquatica* presents promising antioxidant activity and a spectrum of potential biological activities, underscoring its significance as a novel antioxidant candidate for applications in animal nutrition, human medicine, and natural product research in the pharmaceutical and nutraceutical industries.

## 1. Introduction

Medicinal plants, a vital component of traditional and modern healthcare, provide a rich source of bioactive compounds for treating a wide range of diseases [1]. Globally, many plant species are esteemed for their therapeutic properties. Algeria, nestled in the Mediterranean biodiversity hotspot, is renowned for its rich plant species and unique endemism [2,3]. The Algerian flora, particularly in arid and semi-arid regions, has developed remarkable resilience to drought, with species evolving various strategies to thrive in challenging environments [4,5]. This adaptability significantly contributes to the region’s biodiversity and provides an exceptional setting for studying endemism and species distribution, despite facing significant threats from human activity [6,7]. Protecting these plants is, therefore, of particular importance, as Algerian biodiversity includes numerous species with a rich history of medicinal and culinary use that spans centuries [8,9]. This traditional knowledge, passed down through generations, harnesses the health benefits of these plants and supports ongoing research to uncover new pharmaceutical applications [8,9,10,11]. Nevertheless, the chemical makeup and antioxidant benefits of Algerian medicinal plants remain mostly unstudied [12]. A deeper exploration of this phytotherapeutic richness could significantly enhance the value of local biodiversity and reveal new natural therapeutic agents beneficial to human health [13].

The Mentha genus, part of the Lamiaceae family, is renowned in Algeria for its diversity and medicinal value [14,15,16,17,18,19,20,21,22,23,24,25]. Mentha species essential oils are widely valued in both therapeutic and culinary applications [15,17,20]. These plants contain various bioactive compounds, including flavonoids, phenolic acids, and terpenoids, contributing to their antioxidant, antimicrobial, and anti-inflammatory effects [26]. In Algeria, six species of Mentha—*Mentha cervina*, *Mentha longifolia*, *Mentha pulegium*, *Mentha piperita*, *Mentha spicata*, and *Mentha aquatica*—are documented [27], each with a distinct role in Algerian traditional medicine. For example, *Mentha pulegium*, known locally as “Fliou”, is often prepared in infusions to relieve digestive issues such as flatulence, dyspepsia, and intestinal colic due to its carminative and antispasmodic properties [25]. At the same time, *Mentha spicata*, or “Na’na,” is popular in culinary dishes and as a remedy for biliary disorders, menstrual cramps, stomach pain, constipation, gingivitis, and ondotalgies [23]. *Mentha cervina* known locally as “Na’na Ili” is traditionally used to treat digestive disorders, respiratory inflammation, and stomach ailments, as well as a carminative and flea repellent [28]. *Mentha suaveolens* is frequently used for the treatment of various infectious diseases [22]. *Mentha longifolia* and *Mentha piperita* are also used for their respiratory and digestive benefits [29]. Together, these species are essential to Algeria’s phytotherapeutic traditions, embodying the country’s rich biodiversity and cultural heritage [8]. The therapeutic benefits of Mentha species have made them a focal point in local pharmacological research, with the goal of better understanding and applying their properties to meet contemporary health needs.

One of the primary health challenges that medicinal plants like Mentha can help mitigate is oxidative stress. This harmful process results from an imbalance between the production of free radicals and the body’s ability to neutralize them with antioxidant mechanisms, leading to lipids, proteins, and DNA damage [30]. Oxidative stress accelerates cellular aging and plays a significant role in the development of chronic diseases such as cardiovascular disorders, diabetes, neurodegenerative conditions like Alzheimer’s and Parkinson’s, and certain cancers [31,32,33,34]. The antioxidant properties found in Mentha species, enriched with flavonoids, phenolic acids, and terpenoids, make them particularly promising for counteracting oxidative stress and offering hope in the fight against these chronic diseases [26,35,36,37].

In Algeria, Mentha species are actively studied for their antioxidant potential, providing a natural means of reducing oxidative damage and preserving health while reinforcing traditional medicinal knowledge [16,17,19,20,21,24,25,30,38]. Among these, *Mentha aquatica*, commonly known as “Habaq el ma” [39], stands out for its unique combination of culinary and medicinal uses, treating gastrointestinal disorders, pulmonary diseases, and mental conditions such as epilepsy and depression [40,41,42,43]. Studies have shown that *Mentha aquatica* extracts possess numerous pharmacological properties, notably antioxidant, antimicrobial, and anti-obesity activities, with an inhibition of *α*-amylase and pancreatic lipase. It has also shown inhibitory effects on monoamine oxidase (MAO), affinity for GABA-A receptors, and anti-inflammatory properties [44,45,46,47,48,49]. Its essential oil has hepatoprotective, gastrointestinal protective, anti-Alzheimer’s, and anti-cancer effects [50,51,52,53], supported by the presence of triterpenoids, phenolic compounds, and flavonoids, many of which have chemotaxonomic significance [46,54,55,56].

This research aims to deepen the understanding of the phenolic profile of infusion and hydroethanolic extracts from *Mentha aquatica*, evaluating their antioxidant and antidiabetic potential, as well as their capacity to protect human erythrocytes against oxidative stress. By comprehensively identifying and quantifying bioactive compounds by HPLC-ESI-MS/MS and DAD techniques, this study highlights the unique phytochemical diversity of *Mentha aquatica* and its potential impact on health and wellness. These findings could lay the groundwork for utilizing *Mentha aquatica* as a natural food additive, dietary supplement, or ingredient in pharmaceutical formulations. The health-promoting qualities of its phenolic compounds—such as their antioxidant and anti-inflammatory properties—suggest applications beyond diabetes management, extending to the prevention of chronic diseases like cardiovascular conditions, neurodegenerative disorders, and inflammatory diseases. Moreover, the ability of *Mentha aquatica* to inhibit oxidative damage in human cells points to potential uses in skincare products and other cosmeceutical applications, where antioxidant-rich plant extracts are increasingly valued for their protective effects against cellular aging.

As the first study to evaluate the phenolic and bioactive properties of *Mentha aquatica* in Algeria, this research not only enhances local scientific knowledge but also opens avenues for commercial exploration of this plant. The study supports sustainable use of Algeria’s natural biodiversity by identifying new ways to integrate traditional plants into modern health applications. Recognizing the plant’s deep cultural significance, this research may foster innovation in local industries, encourage conservation efforts, and help to preserve traditional knowledge, ultimately paving the way for novel therapeutic and commercial uses that leverage Algeria’s rich plant heritage.

## 2. Materials and Methods

### 2.1. Chemicals

All chemicals used in this study were of analytical grade. Cyanidin 3-*O*-glucoside (Cy3Gluc), cyanidin 3-*O*-rutinoside (Cy3Rut), pelargonidin 3-*O*-rutinoside (Pg3Rut), and peonidin 3-*O*-rutinoside (Pn3Rut) were from Extrasynthese (Genay, France). β-nicotinamide adenine dinucleotide (NADH), phenazine methosulfate (PMS), nitrotetrazolium blue chloride (NBT), 2,2-diphenyl-1-picrylhydrazyl (DPPH), and *α*-glucosidase from Saccharomyces cerevisiae (type I, lyophilized powder) were obtained from Sigma-Aldrich (St. Louis, MO, USA). N-(1-naphthyl) ethylenediamine dihydrochloride, sulfanilamide, 4-Nitrophenyl-*α*-D-glucopyranoside, and sodium nitroprusside dihydrate (SNP), trypsinethylenediaminetetraacetic acid (trypsin-EDTA) solution, 3-(4,5-dimethylthiazol-2-yl)-2,5-diphenyltetrazolium bromide (MTT), dimethyl sulfoxide (DMSO), and sodium nitroprusside dihydrate (SNP) were obtained from Alfa Aesar (Karlsruhe, Germany). Other phenolics and reagents were purchased from Sigma-Aldrich (St. Louis, MO, USA). Methanol and acetonitrile for HPLC (purity ≥ 99.9%) were from Fisher Chemical (Glenfield, Leicestershire, UK). Cell lines were from the American Type Culture Collection (ATCC, Manassas, VA, USA). Water was deionized using a Milli-Q water purification system (Millipore Ibérica, S.A.U., Madrid, Spain).

### 2.2. Plant Material

**Figure 1:**
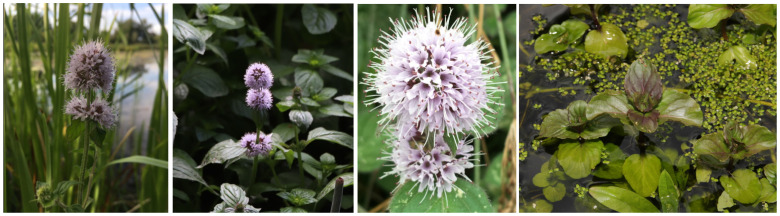
Water Mint (*Mentha aquatica*) in bloom: morphology and ecosystem [57].

The entire flowering plant *Mentha aquatica* was collected in August in the Medjana commune, Oued Sayed, Wilaya of Bordj Bou Arreridj (36°08′ N 4°40′ E). A specimen was identified by the plant taxonomist Prof. Cherchour A. (Faculty of Medicine/Department of Pharmacy, University of Badji Mokhtar-Annaba, Algeria) (Figure 1). Voucher specimens (No. MA-1) were deposited in the herbarium of the laboratory of VALCORE (Valorisation and Conservation of Biological Resources, Biology Department, Faculty of Science, University M’Hamed Bougara, Boumerdes, Algeria).

### 2.3. Infusion and Hydroethanolic Extract Preparation

The aerial parts of *Mentha aquatica* were carefully washed and dried at room temperature, in the dark, for 15 days. Then, the plants were powdered (mean particle size lower than 910 µm) and used for the preparation of infusion and hydroethanolic extracts.

#### 2.3.1. Infusion

Infusion extracts (INF) were extracted according to the method previously reported [58]. One gram of the dried material was boiled with 100 mL (1:100) of water for 5 min, mimicking how it is regularly prepared for human consumption The extract obtained was filtered through a Büchner funnel, frozen and lyophilized and stored in a desiccator in the dark until analysis. Extractions were performed in triplicate. The yield of the INF was calculated gravimetrically as the percentage of the weight of the lyophilized extract to the initial weight of the plant material. The results were expressed as the mean ± standard deviation of three independent extractions.

#### 2.3.2. Hydroethanolic Extract

Hydroethanolic extracts (HE) were performed based on the method described by [59]. The dried material (1 g) was extracted with 100 mL (1:100) of a mixture of ethanol and water (70:30). The preparation was subjected to three extraction steps: a first sonication (30 min), then a maceration (2 h with shaking), and, finally, a second sonication (30 min) at room temperature. The extracts obtained were filtered through a Büchner funnel. (DURAN Group GmbH, Germany). The preparation was then evaporated under reduced pressure (115 mbar), freeze-dried and stored at −20 °C until further analysis. Extractions were performed in triplicate. The yield of the HE was calculated gravimetrically as the percentage of the weight of the lyophilized extract to the initial weight of the plant material. The results were expressed as the mean ± standard deviation of three independent extractions.

### 2.4. Total Phenolic Compound Determination

The total phenolic content in the INF and HE of *Mentha aquatica* was measured using a colorimetric method, with modifications based on the procedure in reference [60]. Briefly, INF and HE extracts were dissolved in methanol at a concentration of 2.5 mg/mL. Then, 50 µL of the methanolic preparations was combined with 450 µL of distilled water and 2.5 mL of Folin–Ciocalteu reagent (0.2 N). After incubating for 5 min at room temperature, 2 mL of a saturated sodium carbonate solution was added, and the total volume was adjusted to 5 mL with distilled water. The mixture was then placed in a 30 °C water bath for 90 min in the dark. Absorbance was measured at 765 nm. Gallic acid was used for the standard curve (range: 50–500 mg/mL; equation: y = 0.0011x + 0.0057, R^2^ = 0.9958). Each measurement was conducted in triplicate, and the results were reported as mean values ± standard deviations, expressed as the molar concentration of gallic acid.

### 2.5. Profiling of Chemical Compounds by High-Performance Liquid Chromatography with Electrospray Ionization and Tandem Mass Spectrometry Detection (HPLC-ESI-MS-MS)

The analysis of phenolic compounds, including flavonoids (e.g., flavanones, glycosides, and methoxyflavones), coumarins, and phenols, as well as non-phenolic compounds such as alkaloids, terpenoids (acyclic monoterpenoids and sesquiterpenes), polyketides, benzoquinones, aromatic esters, and ascorbic acid derivatives, was carried out according to the method of Kerboua et al. [61] on an or-bitrap Thermo q-Exactive mass spectrometer coupled to a Vanquish HPLC. A Kinetex XB-C18 (Phenomenex) with a particle size of 2.6 microns, 100 mm of length, and a diameter of 2.1 mm was used as a column. The mobile phases were 0.1% formic aqueous solution (A) and acetonitrile (B). The gradient program (time (min), % B) was as follows: (0.00, 50); (20.00, 100); (25.00, 100); (35.00, 50). The flow rate was 0.200 mL min^−1^, and the injection volume was 10 µL. The ionization electrospray in positive mode was used. The following analysis parameters were electrospray voltage, −3.8 kV, sheath gas flow rate, 30; auxiliary gas unit flow rate, 10; drying gas temperature, 310 °C; capillary temperature, 320 °C; S-lens and RF level, 55. The acquisition was performed in a mass range from 100 to 1000 a.m.u. An auto MS2 program was used with a fragmentation voltage of 30 V.

### 2.6. Profiling and Quantification of Individual Phenolic Compounds by High-Performance Liquid Chromatography with Diode Array Detector (HPLC-DAD) and High-Performance Liquid Chromatography with Electrospray Ionization and Tandem Mass Spectrometry Detection (HPLC-ESI-MSn)

The identification of bioactive compounds, including phenolic acids, flavonoids, lignans, and other aromatic derivatives, was performed in an Agilent HPLC 1100 series model equipped with a photodiode array detector (model G1315B), a mass detector in series (Agilent Technologies, Waldbronn, Germany), a binary pump (model G1312A), a de-gasser (model G1322A), and an autosampler (model G1313A), based on a method described by Gonçalves et al. [62]. Injections (20 µL) of plant extracts were performed in triplicate. The mass detector was an ion trap spectrometer (model G2445A) with an electrospray ionization interface. It was controlled by LC/MS software (Esquire Control Ver. 6.1. Build No. 534.1., Bruker Daltoniks GmbH, Bremen, Germany). A Nucleosil^®^ 100–5 C18 column (25.0 cm × 0.46 cm; 5 µm particle size waters; Macherey-Nagel, Düren, Germany) was used, and mobile phase was composed of two solvents: eluent A consisted of water/formic acid (99:1, *v*/*v*) and eluent B of acetonitrile.

The solvent system started with 8% of B and reached 15% of B at 25 min, 22% at 55 min, and 40% at 60 min, with a wash-out period of 5 min, and returned to initial conditions afterwards. Mass spectra were acquired with a scan range from *m*/*z* 100 to 1200, and MS parameters were set as follows: capillary temperature was 350 °C, capillary voltage was set at 4 kV, nebulizer pressure was 65.0 psi, and nitrogen flow rate was 11 L/min. The flow rate was 0.8 mL/min during the run, and all gradients were linear. Collision-induced fragmentation experiments were performed in an ion trap using helium as collision gas, with voltage ramping cycles from 0.3 to 2 V. Mass spectrometry data were acquired in a negative ionization mode for non-colored phenolics. MSn was carried out automatically on more abundant fragment ions in MS(n − 1). ChemStation controlled the HPLC system for LC 3D Systems software Rev. B.01.03-SR2 (204) (Agilent Technologies Spain S.L., Madrid, Spain). Phenolic compounds were tentatively identified based on their elution order, retention times, and ultraviolet–visible and mass spectra features as compared to authentic standards analyzed under the same conditions (Table 1) and data available in the literature [62,63,64].

### 2.7. Antioxidant Activity

#### 2.7.1. 2.2-Diphenyl-1-Picrylhydrazil Radical (DPPH^•^)-Scavenging Activity

The effect of *Mentha aquatica* INF and HE extracts on the DPPH^•^ was estimated by a microplate spectrophotometer (Bio-Rad Laboratories; Hercules, CA, USA) method. All dilutions of the samples were previously made in methanol. For each extract, seven different dilutions were prepared. A volume of 25 μL of each concentration was placed in the 96-well microplate, followed by the addition of 200 μL of methanolic DPPH (150 mM). Then, the plates were incubated in the dark for 30 min at room temperature. After that, the absorbance of samples was noted at 515 nm against a blank containing only DPPH solution. The radical scavenging potential of samples was compared with ascorbic acid solutions having different strengths. All experiments were performed in triplicate. The results were expressed as half maximal inhibitory concentration (IC_50_) values (μg/mL). IC_50_ corresponding to the concentration of the sample required to reduce the absorbance of the DPPH solution by 50% was determined from the inhibition curves.

#### 2.7.2. Nitric Oxide (^•^NO) Radical Assay

The ^•^NO scavenging capacity was determined by measuring the accumulation of nitric oxide generated by sodium nitroprusside (SNP) in the preparations with or without the extracts or ascorbic acid (positive control) using the Griess reagent [65]. The two extracts were prepared and serially diluted in phosphate buffer (100 mM, pH 7.4) to give 100 to 1000 μg/mL concentrations. The absorbances were read at 562 nm. The experiments of each extract were performed in triplicate, and the results were expressed as IC_50_ values (μg/mL) calculated by plotting the percentage of inhibition against various concentrations of the extract and determining the concentration required to inhibit 50% of the activity.

#### 2.7.3. Superoxide Radical (O_2_^•−^) Assay

The O_2_^•−^ scavenging capacity was determined as described by Gonçalves et al. [62]. *Mentha aquatica* extracts were tested in a concentration range between 100 and 1000 μg/mL. The reaction was monitored at 560 nm for 2 min, at room temperature. The results were expressed as IC_50_ values (μg/mL), which represent the concentration needed to achieve 50% inhibition.

### 2.8. In Vitro ROO^•−^-Induced Oxidative Damage in Human Erythrocytes

The in vitro ROO^•−^-induced oxidative damage in human erythrocytes was evaluated according to the Gonçalves et al. study [66]. The extracts were dissolved in PBS (1 mg/mL), and six concentrations were prepared. Four experiments were triplicated in each microplate, and the results were expressed as IC_50_ values (μg/mL).

#### 2.8.1. Isolation of Human Erythrocytes

Venous human blood was collected from randomized patients in the Centro Hospitalar of Cova da Beira (Covilhã) by antecubital venipuncture into K3EDTA vacuum tubes. Erythrocytes were isolated based on the procedure described by Gonçalves et al. [62,66].

#### 2.8.2. Inhibition of Hemoglobin Oxidation

The inhibition of hemoglobin oxidation (Hb_ox) was determined by monitoring the lyophilized extracts’ ability to prevent methemoglobin formation [67]. The decomposition of AAPH (dissolved in PBS) is achieved by the water-bath temperature (37 °C), which reacts with oxyhemoglobin, forming methemoglobin. Six dilutions of each extract were prepared with PBS. The sample solution was mixed with erythrocytes solution forming the reaction mixture. Control and blank were performed by replacing the sample with PBS. The reaction mixtures were incubated in a water bath at 37 °C for 30 min, under slow agitation (50 rpm). After incubation, AAPH was added to the mixture (except in the blank), followed by incubation using the same conditions described above for 4 h. The entire volume was transferred to conic Eppendorf’s and centrifuged. The supernatant (300 µL) was placed in a 96-well plate, and the absorbance was read at 630 nm. The results were expressed as IC_50_ values (μg/mL). Five experiments were performed in duplicate. Quercetin was used as a positive control.

### 2.9. Inhibition of Lipid Peroxidation

Lipid peroxidation (LP_OX) in erythrocytes was indirectly assessed by the formation of thiobarbituric-acid-reactive substances (TBARS) [67]. Six different concentrations of infusion and hydroethanolic extracts were dissolved in PBS and mixed with human cell suspension at 37 °C for 30 min with slow agitation (≈50 rpm). After incubation, tert-butyl hydroperoxide (tBHP) was added to the media, which was then further incubated at 37 °C under slow agitation for 30 min. After incubation, the whole content was collected and transferred to conic Eppendorf’s, and trichloroacetic acid (TCA) was added to promote protein precipitation, followed by centrifugation. The reaction of malondialdehyde (MDA) with thiobarbituric acid (TBA) to form TBARS was achieved by inserting the supernatant in a 2 mL conical test tube (with screw cap), followed by the addition of TBA 1% (*w*/*v*). The resulting mixture was heated for 15 min at 100 °C in a water bath. Finally, the test tubes were cooled at room temperature, and the absorbance was measured at 532 nm. The final results were expressed as IC_50_ value (µg/mL). Five experiments were performed in duplicate. Quercetin was used as a positive control.

### 2.10. *α*-Glucosidase Inhibition Assay

The *α*-glucosidase inhibition assay was carried out using a previously established protocol [65]. A total of seven different concentrations of the extract were prepared. For each well, 150 µL of potassium phosphate buffer (19 mM, pH 7.4) was mixed with 50 µL of the sample dissolved in the same buffer and 100 µL of 4-nitrophenyl-*α*-D-glucopyranoside (PNP-G) as the substrate. The control contained only phosphate buffer and PNP-G. The reaction began by adding 25 µL of *α*-glucosidase enzyme to each well, followed by a 10 min incubation at 37 °C. After incubation, the absorbance of the released 4-nitrophenol was measured at 405 nm to assess enzyme activity. Acarbose was used as a positive control. Each experiment was conducted in triplicate, and three independent replicates were performed for accuracy.

### 2.11. Cell Viability Assays

#### 2.11.1. Cell Culture

The N27 and NHDF cells were obtained from the American Type Culture Collection (ATCC; Manassas, VA, USA) and were cultured in 75 cm^2^ culture flasks at 37 °C in a humidified air incubator with 5% CO_2_ [68]. N27 cells were cultured in RPMI 1640 medium with 10% fetal bovine serum (FBS) and 1% antibiotic mixture of 10,000 U/mL penicillin G and 100 mg/mL streptomycin. NHDF cells were cultured in RPMI 1640 medium supplemented with 10% FBS, 2 mM L-glutamine, 10 mM HEPES, 1 mM sodium pyruvate, and 1% of the antibiotic/antimycotic Ab. The medium was renewed every 24 h (N27 cells) to 2 days (NHDF cells) until cells nearly reached the confluence state. When cells reach approximately 90–95% confluence, they are gently detached by trypsinization (trypsin-EDTA solution: 0.125 g/L of trypsin and 0.02 g/L of EDTA). Before each experiment, viable cells were counted in a Neubauer chamber by a trypan-blue exclusion assay and adequately diluted in the appropriate complete cell culture medium. During the assays, NHDF cells were used between passages 22 and 25 and for N27 cells, from 10 to 14.

#### 2.11.2. 3-(4,5-Dimethylthiazol-2-yl)-2,5-diphenyltetrazolium Bromide (MTT) Assay

After reaching a confluence state, cells were trypsinized and counted by the trypan-blue exclusion assay, then seeded with an initial density of 2 × 10^4^ cells/mL (for NHDF) and 1 × 10^5^ cells/mL (for N27) in 96-well culture plates (Nunc, Apogent, Denmark), and left to adhere and grow for 48 h. Subsequently, the medium was removed, and the cells were treated with INF and HE solutions (5 to 1200 μg/mL) in a complete culture medium for 24 h, 48 h, and 72 h. Untreated cells were used as the negative control. Each experiment was performed in quadruplicate and repeated independently at least two times. The in vitro antiproliferative effects were evaluated by the MTT assay. After the incubation period, the medium was removed, and 100 µL of phosphate buffer saline (PBS, NaCl 137 mM; KCl 2.7 mM; Na_2_HPO_4_ 10 mM; and KH_2_PO_4_ 1.8 mM in deionized water with a pH adjusted to 7.4) was used to wash the cells. Then, 100 µL of the MTT solution (5 mg/mL) was prepared in the appropriate serum-free medium and added to each well, followed by incubation for 4 h at 37 °C. The MTT-containing medium was removed, and the formazan crystals were dissolved in DMSO. Then, the absorbance was measured at 570 nm using an xMarkTM microplate spectrophotometer (BIO-RAD Laboratories, Hercules, CA, USA). The values of cell proliferation were expressed as percentages based on the relative absorbance measured in the treated wells versus the control wells.

### 2.12. Statistical Analysis

GraphPad Prism 9 software (GraphPad, La Jolla, CA, USA) was employed to do the statistical analysis, including a one-way analysis of variance (ANOVA) followed by a Tukey multiple comparison test. Student’s *t*-test was utilized to compare two distinct samples, with a significance level of *p* < 0.05. Differences between groups were considered statistically significant at a *p*-value lower than 0.05 (*p* < 0.05).

## 3. Results and Discussion

### 3.1. The Yield and the Total Phenolic Content of Mentha aquatica Extracts

The yield and total phenolic content (TPC) are crucial metrics that provide complementary insights into both the efficiency and quality of extraction methods used for *Mentha aquatica* (Table 2). A higher yield indicates a more efficient extraction process, suggesting that a larger quantity of the plant material has been successfully converted into extract. This is particularly important for ensuring the economic viability of the extraction process in large-scale applications. TPC, on the other hand, is a key indicator of the quality of the extract. Phenolic compounds are well-known for their antioxidant properties, and a higher TPC typically correlates with greater potential health benefits [69]. These compounds play a significant role in neutralizing free radicals, thereby contributing to the therapeutic properties of the plant extract. Therefore, an extraction method that yields a high TPC along with a good yield is considered superior, as it maximizes both the quantity and quality of the extract.

In our study, the INF has a higher extraction yield of 16.210 ± 6.690%, compared to the hydroethanolic extract (HE), which has a yield of 12.113 ± 0.021% (Table 2). This indicates that the infusion process is more effective in extracting a larger amount of overall material from the plant, likely due to the high solubility of many plant constituents in water. However, the higher yield does not necessarily correlate with a significantly higher concentration of specific bioactive compounds, such as phenolics. When we look at the total phenolic content, HE showed a slightly higher value (177.078 ± 4.842 mg GAE/g DW) compared to INF (170.290 ± 8.505 mg GAE/g DW), indicating that the hydroethanolic extraction process has a marginally better capacity to extract phenolic compounds than simple infusion (Table 2). However, the differences are not statistically significant. When comparing our results to other studies, the TPC values obtained for both HE and INF are much higher than those reported by Fidan et al. [70] and Benabdallah et al. [44], where TPC values for ethanolic and hydroethanolic extracts were 43.36 mg GAE/g and 43.21 ± 1.09 mg GAE/g DW, respectively [44,70]. Interestingly, Pereira [51] reported a significantly higher TPC of 307 ± 29 μg/mg for a hydroethanolic extract of *Mentha aquatica*, highlighting the substantial variability in phenolic content depending on the specific extraction conditions, such as solvent concentration, plant material, and extraction protocol [51]. This difference underscores the effectiveness of hydroethanolic extraction for optimizing phenolic recovery, although it also illustrates the variability that can arise from different experimental setups. Additionally, the study by Silva et al. [71] found that for *Mentha spicata*, both the infusion and hydroethanolic extract had relatively close TPC values (38.79 mg/g and 57.92 mg/g, respectively), whereas the decoction showed the highest TPC (77.20 mg/g). This suggests that decoction can extract more phenolic compounds than either infusion or hydroethanolic extraction, regardless of the plant used [71]. Similarly, Tourabi et al. [72] demonstrated that for *Mentha longifolia*, aqueous extracts had the lowest TPC (17.90 ± 0.49 mg GAE/g DW), while hydroethanolic extracts exhibited the highest TPC (23.52 ± 0.14 mg GAE/g DW) [72].

### 3.2. Phytochemical Profiling by HPLC-ESI-MS-MS

The chemical analysis of *Mentha aquatica* extracts reveals a diverse array of bioactive compounds with significant potential for pharmacological applications. A total of 136 compounds were detected in both the infusion (INF) and hydroethanolic extract (HE) using HPLC/ESI/MS/MS (Supplementary Table S1). The identification of the key compounds was achieved by analyzing their MS/MS spectra, supported by our system’s resources and cross-referencing with published studies (Table 3).

#### 3.2.1. Infusion

The compounds identified in INF include 62 diverse chemical compounds, such as nitrogen-containing compounds, phenolics, glycosides, and various esters, reflecting the plant’s rich chemical diversity and significant bioactive potential (Supplementary Table S1). Of particular interest are 10 biologically active compounds (Table 3, C1 to C10): alkaloids (elaeokanine C), two coumarins (5-hydroxycoumarin, 7-methoxycoumarin), two flavonoids (kaempferol 3-*O*-rutinoside and creoside I), one ascorbic acid derivative (6-*O*-acetylascorbic acid), one aromatic ester (vanillyl nonanoate), one benzoquinone derivative (5-*O*-methyl embelin), and one sesquiterpene ketone (farnesylacetone). These compounds were selected based on their known pharmacological activities, structural uniqueness, and relevance to antioxidant, antimicrobial, and anti-inflammatory properties. These compounds represent key categories of bioactive constituents within *Mentha aquatica,* which have demonstrated efficacy in targeting oxidative stress and supporting cellular health. Focusing on these compounds allows for a targeted exploration of *Mentha aquatica*’s therapeutic potential as they provide a representative sample of the plant’s functional diversity and relevance to health-promoting applications.

Kaempferol 3-*O*-rutinoside, detected at a retention time of 8.34 min, has a molecular formula of C_27_H_30_O_15_ with a molecular ion at *m*/*z* 594.1572 (C1, Table 3). The MS/MS spectra reveal key fragment ions at *m*/*z* 287.0544, 449.1071, and 85.0287. The fragment at *m*/*z* 287.0544 corresponds to the aglycone kaempferol, indicating the loss of the rutinoside moiety. The fragment at *m*/*z* 449.1071 represents the loss of a rhamnose sugar unit from the molecule, and the ion at *m*/*z* 85.0287 is likely due to a small fragment from the sugar structure. These fragmentation patterns are characteristic of kaempferol glycosides and confirm the identity of the compound as kaempferol 3-*O*-rutinoside, a flavonoid glycoside known for its antioxidant, antimicrobial, and anti-diabetic properties [73,74].

The analysis of 5-hydroxycoumarin, identified at a retention time of 8.65 min, revealed a molecular ion at *m*/*z* 162.0310, corresponding to its molecular formula C_9_H_6_O_3_ (C2, Table 3). The MS/MS fragmentation pattern showed key ions at *m*/*z* 135.044, 95.049, 89.039, and 63.024. The ion at *m*/*z* 135.044, resulting from the loss of a hydroxyl group, is characteristic of coumarin derivatives and suggests the stability of the core structure during fragmentation. The smaller ions at *m*/*z* 95.049, 89.039, and 63.024 likely represent the breakdown of aliphatic chains or functional groups attached to the coumarin backbone, indicating the stepwise degradation of the molecule. Similarly, 7-methoxycoumarin, observed at a retention time of 10.09 min, exhibited a molecular ion at *m*/*z* 176.0465, consistent with its molecular formula C_10_H_8_O_3_ (C5, Table 3). The MS/MS spectra displayed significant fragments at *m*/*z* 149.023, 121.029, 111.044, and 65.039. The ion at *m*/*z* 149.023 indicates the loss of the methoxy group, confirming its position on the coumarin ring. Further fragmentation of the core structure is reflected by the ion at *m*/*z* 121.029, while the ion at *m*/*z* 111.044 suggests the presence of a stable aromatic fragment. This pattern highlights the characteristic fragmentation of methoxylated coumarins, providing insights into the structural integrity of the molecule.

The identification of coumarins such as 5-hydroxycoumarin and 7-methoxycoumarin in *Mentha aquatica* aligns with previous reports of coumarins being present across various species within the Mentha genus [36,75]. Coumarins are a class of naturally occurring aromatic compounds known for their diverse biological activities, including anticoagulant, anti-inflammatory, and antioxidant properties [76].

6-*O*-acetylascorbic acid, detected at a retention time of 9.44 min, showed a molecular ion at *m*/*z* 218.0393, matching its molecular formula C_8_H_10_O_7_ (C3, Table 3). The MS/MS fragmentation pattern included key ions at *m*/*z* 95.049, 105.045, 77.039, and 51.024. The ion at *m*/*z* 95.049 corresponds to the loss of the acetyl group, leaving the ascorbate core intact. The ion at *m*/*z* 105.045 suggests the presence of a stable fragment associated with the ascorbic acid structure, while the ions at *m*/*z* 77.039 and 51.024 represent smaller, stable fragments. This fragmentation pattern is typical of ascorbic acid derivatives, reflecting their structural features and stability under MS/MS conditions. Interestingly, 6-*O*-acetylascorbic acid has also been reported in *Verbascum betonicifolium*, suggesting that this compound may play a broader role in the antioxidant defense mechanisms of different plant families [77].

Compound 4 (Table 3), detected at a retention time of 9.87 min, exhibited a molecular ion at *m*/*z* 211.1564. The MS/MS spectra revealed product ions at *m*/*z* 55.055, 91.055, 79.055, and 141.070. These spectral characteristics indicate that the compound corresponds to elaeokanine C, an alkaloid with the molecular formula C_12_H_21_NO_2_. This compound was reported as an active compound in *Averrhoa bilimbi* [78].

Creoside I, an acyclic monoterpenoid, exhibits a fragmentation pattern that provides valuable insights into its structural characteristics (C6, Table 3). The ion at *m*/*z* 68.9976 indicates a small, stable aliphatic fragment, possibly representing a simple segment of the monoterpenoid structure. The fragment at *m*/*z* 129.0181 suggests a larger substructure, likely associated with the core backbone of the monoterpenoid, reflecting a key component of the molecule that remains intact during fragmentation. The ion at *m*/*z* 185.0804 may correspond to a significant portion of the monoterpenoid chain, potentially involving a functional group such as an ester or ether bond. Lastly, the fragment at *m*/*z* 255.4873 represents a substantial subunit, possibly involving multiple functional groups such as hydroxyls or ethers, indicating the cleavage of a larger portion of the molecule while retaining its core structure. These fragmentation patterns provide crucial information for understanding the breakdown pathways and structural elements of creoside I, contributing to the elucidation of its detailed molecular composition and potential bioactive properties as an acyclic monoterpenoid. The detection of creoside I in *Mentha aquatica* is significant, especially considering that it has previously been reported in *Rhodiola crenulata*, a plant known for its adaptogenic and medicinal properties [79].

The detection of antiarol (3,4,5-trimethoxyphenol) at a retention time of 10.54 min, with a molecular formula of C_9_H_12_O_4_ and a molecular ion at *m*/*z* 184.0728, sheds light on its phenolic nature (C7, Table 3). The fragmentation ions at *m*/*z* 68.998 and 129.018 point to a stable aromatic ring structure, with the fragment at *m*/*z* 129.018 likely representing the core phenolic group. Previously, it has been reported in *Vitis vinifera*, *Diospyros eriantha,* and *Tarenna attenuata*, [80,81,82].

Vanillyl nonanoate, found at a retention time of 10.79 min, has a molecular formula of C_17_H_26_O_4_ and a molecular ion at *m*/*z* 294.1822 (C8, Table 2). The significant fragments at *m*/*z* 221.1167 and 133.101 provide crucial structural information. The ion at *m*/*z* 221.1167 suggests the loss of the nonanoic acid side chain, while the fragment at *m*/*z* 133.101 corresponds to the vanillyl core structure, which is recognized for its sensory and biological activities. Vanillyl nonanoate is likely to exhibit analgesic and anti-inflammatory effects, as aromatic esters often interact with sensory receptors to alleviate pain and inflammation [83]

Compound 9 belongs to the class of monohydroxy-1,4-benzoquinones, specifically known as 5-*O*-Methyl embelin, where the hydroxy group at position 5 in embelin is substituted with a methoxy group. It was isolated from Lysimachia punctata and Embelia ribes [84,85]. The mass spectrum confirms its presence in the INF of Mentha, showing a retention time (RT) of 11.18 min, with a molecular ion peak at *m*/*z* 308.1977 and fragment ions at *m*/*z* 221.117 and 107.049, suggesting a complex benzoquinone structure. The fragment at *m*/*z* 221.117 likely represents the benzoquinone core, while the fragment at *m*/*z* 107.049 indicates a methoxy-substituted aromatic ring. Benzoquinone derivatives, such as 5-*O*-methyl embelin, have been reported to possess various pharmacological activities, including potent cytotoxic, contraceptive, antimicrobial, antiparasitic, analgesic, and anti-inflammatory effects [86].

Farnesylacetone, observed at a retention time of 12.55 min, has a molecular formula of C_18_H_30_O and a molecular ion at *m*/*z* 262.2287. The fragmentation pattern includes ions at *m*/*z* 105.0702, 95.0858, 81.0703, and 67.0548, reflecting the typical breakdown of sesquiterpene ketones. The fragment at *m*/*z* 105.0702 likely corresponds to the central ketone structure, while the smaller fragments at *m*/*z* 95.0858 and 81.0703 indicate the loss of aliphatic side chains, which is common for terpenoid fragmentation. Sesquiterpene ketones like farnesylacetone are known for their anti-inflammatory and antifungal properties and are often used in traditional medicine and cosmetic products for their therapeutic benefits. This has been reported in *Ononis angustissima* Lam. subsp. *filifolia* Murb, *Artemisia annua* and *Marrubium globosum* subsp. *globosum* [87,88,89].

#### 3.2.2. Hydroethanolic Extract

The compounds identified in the hydroethanolic extract (HE) of *Mentha aquatica* reflect a diverse chemical profile consisting of 63 distinct compounds (Supplementary Table S1), including flavonoids, alkaloids, phenolics, and various esters. Of particular interest are 17 biologically active compounds (Table 3, C11 to C27) grouped into distinct chemical classes such as flavanones, flavones, coumarins, and phenols. Each class contributes uniquely to the plant’s potential pharmacological benefits, particularly in antioxidant, anti-inflammatory, and enzyme-inhibitory activities. Within the flavanones, neoeriocitrin (C11, molecular ion at *m*/*z* 595.1657) and Eriodictyol (C12, molecular ion at *m*/*z* 287.0552) play crucial roles. Neoeriocitrin, in particular, plays a key role with key MS/MS fragments at *m*/*z* 289.0702 and 435.1274. Recent studies suggest that neoeriocitrin may be a promising candidate for the treatment of osteoporosis as its bioactive components have demonstrated the potential to enhance bone health by promoting osteogenic differentiation and inhibiting bone resorption [90].

Eriodictyol, with its key fragmentation ions at *m*/*z* 285.0387 and 135.0439, contributes to the plant’s antioxidant profile by scavenging reactive oxygen species (ROS) [91]. Naringenin-8-*O*-glucoside (C13, molecular ion at *m*/*z* 433.1113), another flavanone, shows potential in inhibiting enzymes such as *α*-glucosidase, making it relevant for diabetes management. Its glycosylated form enhances its bioavailability and increases its effectiveness in controlling postprandial blood sugar levels [92].

In the flavone group, kaempferol (C15, molecular ion at *m*/*z* 285.0394) and scutellarin (C16, molecular ion at *m*/*z* 461.0714) are highly prominent. Kaempferol exhibits anti-inflammatory and anticancer activities, with MS/MS fragments at *m*/*z* 255.0298 and 135.0439 [93]. Scutellarin, a glucosylflavone, displays neuroprotective and anti-inflammatory properties, with key fragmentation ions at *m*/*z* 285.0394, making it beneficial in treating neurodegenerative diseases [94]. Nictoflorin (C14, molecular ion at *m*/*z* 359.1501) adds to the antioxidant and antidiabetic properties of the extract. Its MS/MS fragmentation pattern, including key ions at *m*/*z* 287.0447, reflects its dual role in scavenging ROS and reducing inflammation [95].

Kaempferitrin (C18), classified as a flavonol glycoside, has a retention time of 8.36 min and a molecular formula of C_27_H_30_O_15_. Its key MS/MS fragments appear at *m*/*z* 287.0552, 433.1113, and 285.0394. Known for its antioxidant and anti-inflammatory properties, kaempferitrin contributes to the extract’s capacity to manage oxidative stress [96].

Persicogenin (C21), a 3′,5-dihydroxy-4′,7-dimethoxyflavanone, shows a retention time of 10.17 min with a molecular formula of C_17_H_16_O_6_, and MS/MS fragments at *m*/*z* 315.0863 and 287.0552. This compound has been reported as an anti-mutagenic, anticancer, and anti-mycobacterial agent [97]. Amorphaquinone (C22) is identified with a retention time of 10.20 min and a molecular formula of C_18_H_18_O_7_. Its MS/MS fragments are at *m*/*z* 345.0984 and 271.0595. It has antiparasitic and antimicrobial properties [98]. Combretol (C26), a pentamethoxyflavone, known for its leishmanicidal activity has a retention time of 11.42 min and a molecular formula of C_19_H_18_O_7_, with MS/MS fragmentation at *m*/*z* 357.0979 and 329.0654 [99].

The coumarin family is represented by 4-hydroxycoumarin (C17, molecular ion at *m*/*z* 163.0383) and 7-methoxycoumarin (C20, molecular ion at *m*/*z* 195.0393). Both compounds contribute anti-inflammatory properties and show strong antioxidant activities, protecting biological tissues from oxidative stress [100].

Elaeocyanidin (C25), a leucoanthocyanidin with a molecular ion at *m*/*z* 359.1125, exhibits a series of characteristic fragmentation ions that help confirm its structure. The fragment at *m*/*z* 227.0545 likely corresponds to a cleavage of part of the flavonoid core, indicating the loss of a specific structural group. The ion at *m*/*z* 197.0078 suggests further fragmentation within the polyphenolic backbone, while *m*/*z* 169.0129 is commonly associated with the formation of a stable aromatic ring structure. Finally, the fragment at *m*/*z* 215.0182 indicates another cleavage that may represent the loss of specific hydroxyl or methoxy groups, consistent with the structural characteristics of leucoanthocyanidins.

Retusin, a tetramethoxyflavone (C27), is another significant compound. With a molecular ion at *m*/*z* 357.0979, it exhibits key MS/MS fragments at *m*/*z* 329.065, 311.0546, 197.0079, and 169.0129. The fragment at *m*/*z* 329.065 corresponds to the loss of a methoxy group (–OCH_3_), while the ion at *m*/*z* 311.0546 indicates further cleavage within the flavonoid structure. The fragment at *m*/*z* 197.0079 likely results from the breakdown of the flavonoid core, and the ion at *m*/*z* 169.0129 suggests the formation of a stable aromatic structure, further confirming the presence of a highly substituted flavonoid. Retusin is known for its potent antioxidant properties, protecting cells from oxidative damage and contributing to overall cellular health [101].

### 3.3. Profiling and Quantification of Individual Phenolic Compounds by HPLC-DAD and HPLC-ESI-MSn

Phenolic compounds present in *Mentha aquatica* were analyzed in the plant dried-treated (by methanol 50% + formic acid 1%) as well as in both infusion (INF) and hydroethanolic (HE) extracts. They were then quantified using HPLC-DAD-ESI-MSn (Table 4). The results show significant differences in the quantities and types of compounds extracted depending on the method used (Compounds C1 to C30). The HE proves particularly effective for isolating certain phenolic compounds such as dimethyl caffeic acid and its derivatives. Specifically, dimethyl caffeic acid derivatives (C17) are found at a concentration of 3100.73 ± 13.78 µg/g in the HE, while dimethyl caffeic acid hexose (C19) reaches 4744.83 ± 34.00 µg/g of extract. These high concentrations suggest that the hydroethanolic solvent is more suitable for extracting these compounds, likely due to their higher solubility in this solvent mixture, which combines the polar properties of both water and ethanol [102,103,104].

In contrast, ferulic acid derivatives also showed a strong preference for hydroethanolic extraction, with concentrations reaching 98,324.02 ± 783.94 µg/g for ferulic acid derivative 2 (C22), 70,297.35 ± 392.71 µg/g for ferulic acid derivative 1 (C21). These results indicate that the HE is superior for isolating these specific molecules, which are known for their antioxidant and anti-inflammatory properties [105]. The absence of these compounds in the INF suggests that water alone is not as efficient in breaking down plant cell walls or in dissolving these compounds, possibly due to their lower polarity or stability in purely aqueous solutions [102,106]. However, the efficiency of extracting these bioactive compounds is not solely dependent on the choice of solvent. Several factors can significantly influence the extraction yield and composition of the extracts [107]. The drying method and temperature are particularly crucial as they can alter the chemical structure and stability of phenolic compounds [18]. High temperatures can lead to the degradation of sensitive compounds, while freeze-drying can help to preserve the integrity of these bioactive molecules [54]. Similarly, extraction time, especially when using techniques such as sonication, plays a vital role [108]. Optimizing sonication time is essential to balance between maximizing yield and preserving compound integrity, as prolonged sonication can cause the degradation of certain compounds due to heat and ultrasound energy [109].

In our study, certain phenolic compounds (Table 4)—syringic acid (C1), apigenin-7-*O*-rutinoside (C13), hesperetin-7-*O*-rutinoside (C15), dimethyl caffeic acid (C17), feruloyl derivative (C20), quercetin (C28), and quercetin derivative (C29)—were quantified only in the *Mentha aquatica* samples treated with 50% methanol and 1% formic acid (A). The absence of these compounds in INF and HE extracts suggests that the methanol-based treatment was more effective at extracting these particular phenolic compounds, likely due to the solvent’s specific polarity and ability to disrupt plant cell walls more effectively than water or hydroethanolic mixtures alone [110]. This selective extraction highlights the importance of solvent choice in maximizing the yield of certain phenolics as methanol may better solubilize less polar phenolic compounds like those mentioned [111]. Interestingly, certain phenolic compounds show a greater affinity for the aqueous environment of the INF. Galloyl derivative (C26), for example (Table 4), is found at a concentration of 35,273.99 ± 45.58 µg/g but is absent in the HE. This suggests that water is a better solvent for these compounds, possibly due to their higher hydrophilicity [112]. Similarly, quinic acid (C30), quantified at 21,151.90 ± 83.26 µg/g (Table 4), and the *p*-hydroxybenzoic acid derivative at 24,588.37 ± 103.65 µg/g in the INF extract (Table 4) indicate that these compounds are more efficiently extracted in a purely aqueous environment [102].

Additionally, the geographical origin and habitat of *Mentha aquatica* significantly impact its phytochemical composition [113]. Environmental factors such as soil type, altitude, climate, and exposure to biotic and abiotic stressors can influence the concentration and diversity of secondary metabolites in the plant [114]. Plants grown in regions with high sunlight exposure or nutrient-rich soils may produce higher levels of certain phenolic compounds as a defense mechanism against environmental stressors. Furthermore, the plant’s habitat can also affect the balance between primary and secondary metabolites, potentially altering its medicinal properties and effectiveness [115].

The total phenolic content, represented by the sum of all identified compounds, is higher in the HE (184,347.57 µg/g) compared to the INF (96,593.60 µg/g) and Mentha-treated (A) sample (21,403.55 µg/g). This significant difference underscores the broader extraction capacity of the hydroethanolic solvent, capable of isolating a wider range of phenolic compounds, including those that are less polar and thus poorly soluble in water alone. These variations underscore the importance of carefully selecting the extraction method and considering the environmental factors influencing plant composition to optimize the yield and efficacy of phenolic compounds in *Mentha aquatica* extracts [103].

### 3.4. Biological Activities

The biological activities of medicinal plants, namely, their antioxidant, and anti-inflammatory properties, are attracting increasing interest due to their therapeutic potential in the prevention and treatment of various diseases [116]. Bioactive plant secondary metabolites are abundantly present in medicinal plants and possess remarkable therapeutic potential. Oxidative stress and drug resistance are key contributors to contemporary health issues such as diabetes, atherosclerosis, cardiovascular disorders, cancer, and inflammation [117]. The bioactive compounds present in plant extracts, such as flavonoids, polyphenols, and alkaloids, play a crucial role in neutralizing free radicals, modulating enzymatic activity, and protecting against oxidative damage. Evaluating these activities allows for a better understanding of the mechanisms of the actions of these extracts and the identification of those with significant beneficial properties, potentially suitable for pharmacological applications [118].

In this study, the INF and HE of *Mentha aquatica* were analyzed to evaluate their antioxidant, antiradical, and antidiabetic potential, as well as their ability to protect human erythrocytes against oxidative stress in comparison with quercetin and ascorbic acid as reference standards. The results are summarized in Table 5.

#### 3.4.1. Antioxidant Activity

**Figure 2:**
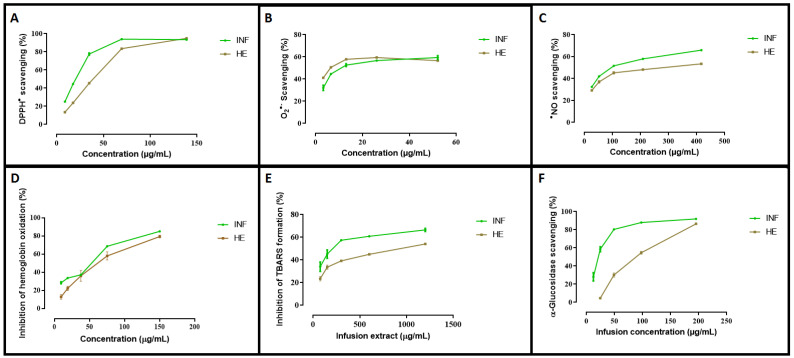
Scavenging activity against DPPH^•^ radical (**A**), superoxide anion (O_2_•⁻) (**B**), radical NO• (**C**), and the inhibition of hemoglobin oxidation (**D**), lipid peroxidation (**E**), and *α*-glucosidase activity (**F**) of *Mentha aquatica* infusion (INF) and hydroethanolic (HE) extracts. Values expressed as mean ± SD; n = 3.

The antioxidant activities of plant extracts are typically evaluated using various assays, each targeting different reactive oxygen species (ROS) or reactive nitrogen species (RNS) to provide a comprehensive understanding of the extract’s ability to combat oxidative stress [119]. The most commonly used assays include 2,2-diphenyl-1-picrylhydrazyl (DPPH^•^), nitric oxide (^•^NO), and superoxide radical scavenging assays (O_2_^•−^). These assays measure the extract’s capacity to neutralize specific free radicals, thus preventing cellular damage associated with oxidative stress. The results of this study demonstrate varying levels of antioxidant activity for the INF and HE of *Mentha aquatica* across different assays, highlighting the complex interplay between extraction methods, bioactive compound concentration, and antioxidant efficacy (Table 5 and Figure 2).

a. DPPH Radical Scavenging Activity

The DPPH assay is widely used to evaluate the free radical scavenging ability of antioxidants in plant extracts. This assay measures the capacity of the extract to donate an electron or hydrogen to stabilize the DPPH^•^ [119].

In our study, all extracts exhibited antioxidant activity in a concentration-dependent manner (Figure 2A). INF shows superior activity with an IC_50_ of 20.353 ± 0.563 µg/mL, compared to the HE, which has a significantly higher IC_50_ of 38.875 ± 0.693 µg/mL (Table 5). Despite the HE being more concentrated in bioactive compounds according to the previous phytochemical analysis, its lower efficacy in the DPPH^•^ test could be due to the specific composition and interactions of compounds in the INF that enhance its antioxidant properties. The standard used in this assay, ascorbic acid, exhibits a much lower IC_50_ of 6.720 ± 0.123 µg/mL, confirming its superior free radical scavenging ability. This result aligns with the determined in previous studies as ascorbic acid is a well-known potent antioxidant [120]. When compared to other studies on species of the Mentha genus, the IC_50_ for DPPH^•^ radical scavenging activity is 19.98 ± 0.91 µg/mL for the hydroethanolic extract (70/30 *v*/*v*) of *Mentha pulegium*, suggesting a slightly higher antioxidant activity for this species [121]. Another study by Boualam et al. [122] showed that the aqueous extract of *Mentha rotundifolia* has an IC_50_ value of 26.47 µg/mL in the DPPH assay and exhibits neuroprotective properties by reducing the effects of hydrogen peroxide (H_2_O_2_) in rats. The high content of kaempferol glucuronide is cited as being responsible for these beneficial effects [122]. Such comparisons highlight the variations in antioxidant potential among different Mentha species, which can be attributed to differences in the phytochemical composition of each extract.

b. Superoxide Radical Scavenging Activity

The superoxide radical scavenging (SO) assay evaluates the extract’s ability to neutralize superoxide anions, which are highly reactive oxygen species that can cause oxidative damage to cells and tissues. Superoxide anions are typically generated as a byproduct of cellular respiration and are involved in various physiological and pathological processes. If not adequately neutralized, these radicals can lead to oxidative stress, which is linked to numerous chronic diseases, including cardiovascular disorders, neurodegenerative diseases, and cancer [119]. The mechanism of action for superoxide radical scavenging involves the extract donating an electron or hydrogen atom to the superoxide anion (O_2_⁻•), thereby neutralizing it and converting it into a less reactive species, such as hydrogen peroxide (H_2_O_2_). This process prevents the superoxide anion from reacting with other molecules, such as nitric oxide (NO), to form more harmful species like peroxynitrite (ONOO⁻). Concerning *Mentha aquatica* extracts, they showed a slight concentration-dependent behavior (Figure 2B). The INF again outperforms the HE, with an IC_50_ of 96.484 ± 3.251 µg/mL compared to 281.417 ± 21.019 µg/mL for the HE (Table 5). The significant difference between these two extracts indicates that the compounds present in the INF are more effective in scavenging superoxide radicals. The SO assay specifically evaluates the ability to neutralize superoxide anions, which are highly reactive species contributing to oxidative stress.

The fact that the INF performs better despite the HE’s higher concentration of bioactive compounds suggests that the qualitative nature of the compounds and their synergistic interactions play a crucial role. Ascorbic acid, used as a reference standard in this test, has an IC_50_ of 142.4228 ± 6.356 µg/mL, which places it between the two extracts in terms of efficacy (Table 5). This indicates that while ascorbic acid is effective, the INF of *Mentha aquatica* is even more potent in neutralizing superoxide radicals. Previous studies have shown that the superoxide radical scavenging activity of different solvent fractions of *Mentha spicata* was investigated, demonstrating that the ethyl acetate and aqueous fractions of the ethanol extract exhibited higher superoxide radical scavenging activity compared to other fractions [123]. Another study examined the effect of heat stress on the essential oil composition and antioxidant enzyme activity, including SO, in *Mentha piperita* and *Mentha arvensis*. It was found that heat stress significantly affected the SO activity in both species, and the use of salicylic acid and melatonin helped to alleviate oxidative stress, thereby enhancing the SO activity [124]. In addition to these findings, research on other Mentha species, such as *Mentha longifolia*, has shown that different extraction methods and plant parts can result in varying levels of SO activity. For instance, the essential oil of *Mentha longifolia* has demonstrated significant superoxide radical scavenging capacity, attributed to its rich phenolic and flavonoid content [125].

Furthermore, the environmental conditions in which these plants are grown play a crucial role in their antioxidant potential. A study conducted on six wild Mentha species from Algeria highlighted the influence of geographical and climatic factors on the total phenolic content and antioxidant activity, suggesting that these external conditions can significantly modulate the bioactive properties of the extracts [44]. Overall, these studies emphasize that the antioxidant efficacy of Mentha extracts is not only dependent on the type of solvent used for extraction but also on the environmental conditions, plant species, and the specific part of the plant used. Such factors must be considered when evaluating the potential therapeutic applications of Mentha extracts, particularly in the context of managing oxidative stress-related conditions.

c. Nitric Oxide (NO) scavenging activity

Nitric oxide (NO) scavenging activity refers to the ability of an extract or compound to neutralize nitric oxide radicals. Nitric oxide is a reactive nitrogen species involved in various physiological processes such as vasodilation, neurotransmission, and immune response. However, excessive production of NO can lead to the formation of peroxynitrite (ONOO⁻) when it reacts with superoxide anions (O_2_⁻•), contributing to oxidative stress and tissue damage, which are associated with conditions like inflammation, cancer, and neurodegenerative diseases [119].

In our study, the INF and HE show comparable activities, with IC_50_ values of 11.073 ± 1.237 µg/mL and 12.73 ± 0.123 µg/mL, respectively (Figure 2C and Table 5). The lack of a statistically significant difference between the two suggests that both extracts possess similar capabilities in neutralizing peroxynitrite radicals. Despite the higher concentration of bioactive compounds in the HE, its performance is nearly equivalent to that of the INF, indicating that the specific compounds effective in this assay are present in comparable amounts or that their reactivity is similar in both extracts. Ascorbic acid, on the other hand, has an IC_50_ of 112.567 ± 8.407 µg/mL, indicating much lower efficacy in this test (Table 5). These results are consistent with findings in other studies where different Mentha species, such as *Mentha spicata* and *Mentha longifolia*, have demonstrated varying degrees of NO scavenging activity depending on the extraction method and solvent used. For *Mentha spicata*, the IC_50_ for NO scavenging was reported to be 210.6 ± 7.7 µg/mL, which demonstrates moderate effectiveness in neutralizing NO radicals at relatively low concentrations [126]. In the case of *Mentha longifolia*, different extracts exhibited varying degrees of NO scavenging activity. The hexane extract of *Mentha longifolia* showed the highest potency with an IC_50_ of 0.010 ± 0.002 mg/mL, followed by the essential oil (IC_50_ = 0.032 ± 0.002 mg/mL) and the methanol extract (IC_50_ = 0.052 ± 0.004 mg/mL) [127]. These results highlight that the choice of solvent can significantly influence the efficiency of NO scavenging, with some solvents extracting more potent compounds than others [54]. The similarity in the NO scavenging activities of INF and HE in our study suggests that either method could be effectively used for obtaining extracts with strong antioxidant properties. However, the choice between them might depend on other factors, such as the desired profile of bioactive compounds, the extraction efficiency, and the intended application.

#### 3.4.2. Protective Effects of *Mentha aquatica* Extracts Against Oxidative Damage in Human Blood Erythrocytes

Plant extracts protect human blood erythrocytes from oxidative damage through mechanisms that inhibit hemoglobin oxidation and lipoperoxidation [128]. These effects are attributed to a wide range of bioactive compounds present in the extracts, including flavonoids, phenolic acids, and other potential antioxidants. Flavonoids and phenolic acids, for example, are known to neutralize reactive oxygen species (ROS), preventing the oxidation of hemoglobin into methemoglobin, which would otherwise impair its oxygen-carrying capacity [129]. Additionally, these compounds can interrupt the chain reactions that lead to lipid peroxidation in cell membranes, maintaining erythrocyte integrity and function. By scavenging free radicals and chelating metal ions that catalyze oxidative reactions, plant extracts help to preserve the structural stability of erythrocytes and protect against hemolysis, highlighting their potential therapeutic use in conditions associated with oxidative stress [128].

The protective effects of plant extracts against oxidative damage in human blood erythrocytes can be evaluated through assays like hemoglobin oxidation (Hb_ox) and lipoperoxidation (LP_ox) tests. These tests are crucial in understanding how plant-derived antioxidants can mitigate oxidative stress, which is known to damage cellular structures, including lipids, proteins, and DNA.

For hemoglobin oxidation (Hb_ox), the INF displays greater efficacy with an IC_50_ of 52.638 ± 0.168 µg/mL, compared to the HE, which has a higher IC_50_ of 128.711 ± 13.274 µg/mL, indicating a significantly lower effectiveness (Figure 2D and Table 5). This statistically significant difference suggests that the INF is more potent in preventing hemoglobin oxidation, potentially due to the presence of more effective compounds or a more favorable composition for this specific activity. Quercetin, with an even higher IC_50_ of 369.701 ± 18.185 µg/mL, is much less effective at inhibiting hemoglobin oxidation compared to *Mentha aquatica* extracts (Table 5). This shows that the combination and synergy of bioactive compounds in the INF and HE are more suited to scavenging the specific reactive oxygen species (ROS) involved in hemoglobin oxidation.

In the lipoperoxidation (LP_ox) test, which measures the ability to inhibit lipid peroxidation, the INF again shows superior performance with an IC_50_ of 267.662 ± 15.827 µg/mL, significantly outperforming the HE, which has an IC_50_ of 490.611 ± 25.305 µg/mL (Figure 2E and Table 5). This significant difference indicates that the INF is more effective in preventing lipid peroxidation, suggesting that the compounds extracted through INF are better at protecting lipids from oxidative damage. However, quercetin exhibits an extremely low IC_50_ of 2.607 ± 0.064 µg/mL, demonstrating exceptionally strong antioxidant activity in preventing lipid peroxidation (Table 5).

The observed differences in the effectiveness of *Mentha aquatica* extracts in preventing hemoglobin oxidation and lipid peroxidation can be attributed to the nature and interactions of their bioactive compounds. Hemoglobin oxidation occurs when reactive oxygen species (ROS), such as superoxide anions (O_2_⁻•) or hydrogen peroxide (H_2_O_2_), react with hemoglobin, impairing its oxygen-carrying capacity by converting it to methemoglobin [130]. The significantly lower IC50 value of the INF compared to the HE suggests that water-soluble compounds in the INF are more effective at neutralizing ROS and chelating transition metal ions like iron, which catalyze the formation of more harmful radicals like hydroxyl radicals (•OH) through the Fenton reaction [129]. This dual mechanism highlights the superior antioxidant potential of the INF extract.

When comparing these results with other species, such as *Mentha pulegium*, *Salvia moorcroftiana*, and *Ocimum sanctum*, distinct differences in efficacy against oxidative damage can be observed [121,131]. The hydroethanolic extract of *Mentha pulegium* demonstrated significant protective effects against AAPH-induced oxidative damage to erythrocyte membranes, with an IC_50_ of 129.52 ± 2.15 µg/mL. While effective, this value is notably higher than that of the *Mentha aquatica* INF (IC_50_ = 52.638 ± 0.168 µg/mL), suggesting that INF is more potent in preventing oxidative damage, likely due to the presence of more efficient water-soluble antioxidants [121]. Hydrolyzed extracts of *Salvia moorcroftiana* and *Ocimum sanctum* showed significantly higher IC_50_ values compared to the INF of *Mentha aquatica*, highlighting its superior protection against hemoglobin oxidation [131]. This effectiveness likely results from the preservation of antioxidant compounds during the infusion process. Lipid peroxidation, caused by ROS attacking polyunsaturated fatty acids in membranes, leads to oxidative damage [132]. The INF outperformed the HE in the LP_ox test, suggesting that its bioactive compounds are better at stabilizing lipid peroxyl radicals and halting oxidative chain reactions [133]. In addition, quercetin demonstrated even greater potency due to its strong radical-scavenging abilities, though *Mentha aquatica* extracts still provide substantial antioxidant protection despite being less effective than pure quercetin or other plants. In the study of Spréa et al. [134], the extracts from *Origanum vulgare*, *Thymus vulgaris*, *Ocimum basilicum*, *Salvia officinalis*, *Melissa officinalis*, and *Matricaria chamomilla* exhibited exceptional antioxidant activity in TBARS assays, with IC_50_ values under 26 µg/mL. This indicates their strong capacity to inhibit lipid peroxidation at very low concentrations, which is likely due to their high content of phenolic compounds and flavonoids that efficiently neutralize lipid peroxyl radicals. Another study of Barros et al. [135] reported remarkably low IC_50_ values for TBARS inhibition in brain homogenates for *Origanum vulgare* (0.01 ± 0.00 mg/mL), *Glechoma hederacea* (0.11 ± 0.01 mg/mL), and *Thymus mastichina* (0.43 ± 0.02 mg/mL) [135]. The difference could be attributed to the distinct phytochemical compositions of these plants, where the concentration and type of phenolic compounds, flavonoids, and other bioactive molecules play a crucial role in their antioxidant efficacy [135].

#### 3.4.3. *α*-Glucosidase Inhibitory Activity

Diabetes is a chronic condition characterized by high blood sugar levels, often managed by inhibiting A-GLUC, which slows the breakdown of carbohydrates into glucose. This helps reduce postprandial blood sugar spikes. Natural plant extracts show promising A-GLUC inhibitory activity, offering a complementary approach to conventional treatments. These natural inhibitors not only help to control blood glucose but also provide additional health benefits due to their antioxidant properties.

In this study, the hydroethanolic extract (HE) showed significantly lower inhibitory activity, with a high IC_50_ of 354.179 µg/mL, compared to the infusion extract (INF), which had an IC_50_ of 20.917 µg/mL (Figure 2F and Table 5). This significant difference suggests that the INF extract, with its high inhibitory activity, could be a key component in the development of more effective antidiabetic treatments. By inhibiting the A-GLUC enzyme, a key player in breaking down carbohydrates into glucose, the INF extract could potentially revolutionize the management of blood glucose levels after meals. Additionally, the standard used in this test, acarbose, has an IC_50_ of 17.017 µg/mL, indicating slightly higher efficacy than the INF extract (Figure 2F and Table 5).

A-GLUC inhibitory activities were also previously reported for the methanol extract of *Mentha pulegium* from Turkey, with IC_50_ values of 20.38 µg/mL, demonstrating strong inhibition [74]. This is closely comparable to the INF of *Mentha aquatica* in the present study, indicating a similar potency in inhibiting A-GLUC activity. When compared to other studies, the root extracts of *Mentha rotundifolia* from the Mila region (IC_50_ = 17.21 ± 0.11 µg/mL) showed even higher A-GLUC inhibition than both the INF and methanol extracts of *Mentha pulegium* [74]. However, the aerial parts of *Mentha rotundifolia* had much lower inhibitory activity, with IC_50_ values ranging from 107.176 ± 1.49 µg/mL to 181.253 ± 3.02 µg/mL, which are still more effective than the HE of *Mentha aquatica* [74]. Moreover, the ethyl acetate fraction of *Mentha pulegium* demonstrated an IC_50_ of 61.85 ± 1.69 µg/mL, which, while effective, is not as potent as the INF of *Mentha aquatica* or the methanol extract of *Mentha pulegium* from Turkey [74,136]. This suggests that the choice of extraction solvent and method significantly influences the concentration and efficacy of active compounds in inhibiting A-GLUC.

The statistically significant difference between the extracts emphasizes the superior performance of the INF in inhibiting A-GLUC activity. These results can be attributed to several mechanisms. The INF process typically extracts a higher concentration of bioactive compounds that can effectively bind to the enzyme’s active site, preventing it from interacting with and hydrolyzing carbohydrate substrates. Indeed, several experiments have reported high correlation between phenolic contents in plant extracts and A-GLUC inhibitory activities [137]. Additionally, the specific combination of compounds in the INF may exhibit synergistic effects, enhancing their collective inhibitory activity. This synergy could increase the binding affinity of these molecules for the enzyme, making the inhibition more potent compared to the HE, where such interactions might be less pronounced. Furthermore, some components in the INF may induce conformational changes in A-GLUC, altering its structure and reducing its catalytic efficiency. This mechanism, known as non-competitive inhibition, can significantly reduce the enzyme’s activity even when substrate levels are high. The water-based extraction method used in the INF may also preserve or enhance the bioavailability and solubility of these inhibitory compounds, allowing them to interact more efficiently with the enzyme. In contrast, the HE may contain less soluble or less bioavailable inhibitory molecules, resulting in lower efficacy.

### 3.5. Cytotoxicity of Mentha Extracts

#### 3.5.1. Normal Human Dermal Fibroblasts (NHDFs)

**Figure 3:**
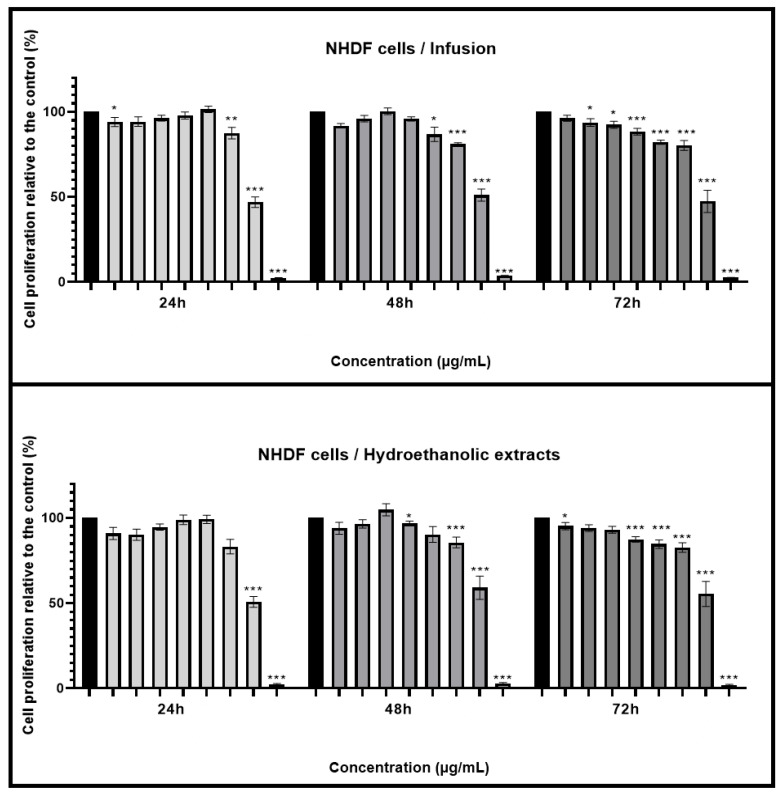
Effect of infusion and hydroethanolic extract on NHDF viability after extracts exposure, assessed by MTT reduction. In the figure, viability is represented by shades of gray, which darken progressively over time (24 h, 48 h, 72 h). Values show mean ± SEM. One-way ANOVA was used to determine statistical significance in NHDF cells compared to control (black bar). Two-way ANOVA was used in NHDF to compare the two extracts in different concentrations (9.375, 18.75, 37.5, 75, 150, and 300 µg/mL). (* *p* < 0.05, ** *p* < 0.01, and *** *p* < 0.0001).

Figure 3 demonstrates the effects of various concentrations of *Mentha aquatica* infusion (INF) and hydroethanolic extract (HE) on the proliferation of NHDF cells (normal human dermal fibroblasts) across 24, 48, and 72 h time points. The tested concentrations ranged from 9.375 µg/mL to 1200 µg/mL (Figure 3). The results illustrate how the cell proliferation relative to control changes as the concentration and exposure time increase, revealing both the cytotoxic and proliferative potential of these extracts.

At 24 h, both the infusion and the hydroethanolic extract show an initial increase in cell proliferation at lower concentrations (9.375 to 150 µg/mL). The highest proliferation is observed around 75 µg/mL for both extracts. However, as the concentration rises beyond 300 µg/mL, a noticeable decline in cell viability occurs. At the highest concentrations (600 to 1200 µg/mL), cytotoxicity is significant, with the extracts inhibiting cell proliferation almost completely (Figure 3). After 48 h, the trend remains consistent: low to moderate concentrations (up to 150 µg/mL) promote cell growth, while concentrations above 300 µg/mL begin to show cytotoxic effects. The extracts exhibit similar behavior, with higher concentrations increasingly inhibiting proliferation. Cytotoxicity becomes even more evident at 600 µg/mL and beyond (Figure 3). By 72 h, the cytotoxic effects of both the infusion and the hydroethanolic extract are even more pronounced. While the lower concentrations still promote cell proliferation, the highest concentration (1200 µg/mL) causes a near-complete inhibition of cell growth, indicating strong cytotoxicity with prolonged exposure (Figure 3).

Based on the IC_50_ values, a clear difference in cytotoxicity can be observed. The HE consistently shows lower IC_50_ values across all time points, indicating higher cytotoxicity compared to the INF. Specifically, at 24 h, the IC_50_ of the HE is 560.3 ± 14.42 µg/mL, which decreases significantly to 449.5 ± 15.56 µg/mL at 48 h before slightly increasing to 471.2 ± 21.45 µg/mL at 72 h. This reduction in IC_50_ over time indicates that the HE becomes more cytotoxic with prolonged exposure, particularly at the 48 h mark. Hydroethanolic extraction pulls a broader range of compounds, including both polar and non-polar substances like flavonoids, phenolic acids, terpenoids, and other secondary metabolites. These compounds are present in higher concentrations in the HE and contribute to its stronger biological activity. However, at elevated doses, the same compounds can disrupt cell function and lead to cytotoxicity. Some of these potent compounds may affect critical cellular pathways, increasing oxidative stress or inducing apoptosis, which explains the stronger cytotoxic effects observed with the HE at higher concentrations [138].

In contrast, the IC_50_ values for the INF remain more stable across time points, with 586.1 ± 15.87 µg/mL at 24 h, 605.1 ± 23.68 µg/mL at 48 h, and 599 ± 26.56 µg/mL at 72 h. These values suggest that the cytotoxic effects of the INF do not increase significantly over time, making it a less aggressive option compared to the HE. The relatively stable IC_50_ values for the INF indicate that it maintains a more consistent and milder impact on cell viability, particularly over extended periods.

Overall, the data suggest that while both extracts exhibit some level of cytotoxicity, the HE is considerably more potent, especially at longer exposures. The INF, on the other hand, shows a more moderate cytotoxic profile, potentially making it a safer choice for therapeutic applications that require lower toxicity. The choice between the HE and the INF would, therefore, depend on the intended use and the desired balance between efficacy and safety, with the INF offering a more stable and less toxic alternative.

#### 3.5.2. N27 Dopaminergic Cells

**Figure 4:**
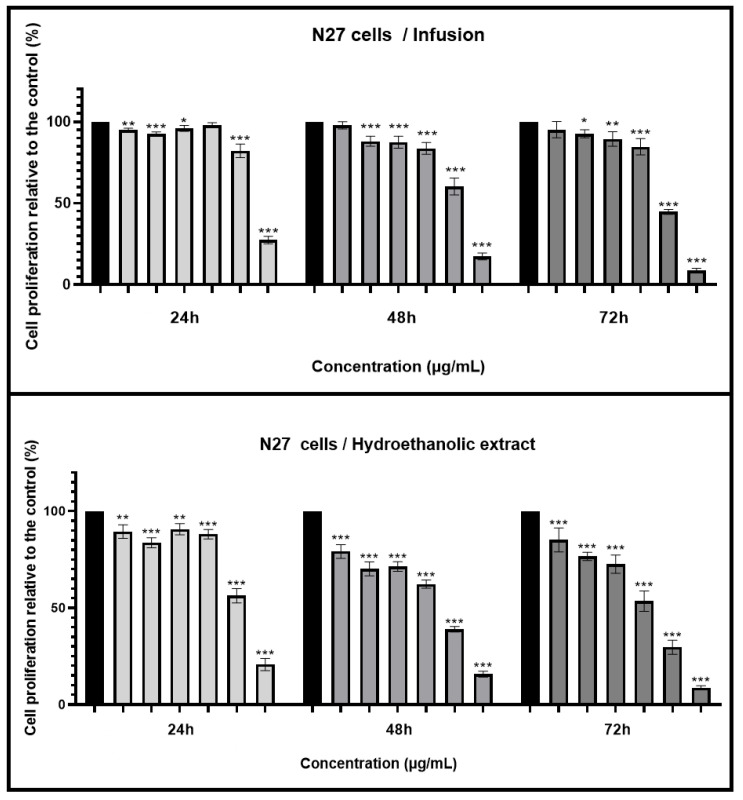
Effect of infusion and hydroethanolic extract on N27 viability after 24 h, 48 h, and 72 h of exposure, assessed by MTT reduction. Values show mean ± standard deviation performed in triplicate. One-way ANOVA was used to determine statistical significance in N27 cells compared to control (black bar). Two-way ANOVA was used in N27 to compare the two extracts in different concentrations (9.375, 18.75, 37.5, 75, 150, and 300 µg/mL). (* *p* < 0.05, ** *p* < 0.01, and *** *p* < 0.0001).

The effects of *Mentha aquatica* INF and HE on N27 dopaminergic cells were evaluated at concentrations ranging from 9.375 to 300 µg/mL over 24, 48, and 72 h (Figure 4). Both extracts exhibited dose- and time-dependent cytotoxicity, with the hydroethanolic extract showing stronger cytotoxic effects overall.

For the INF, cell proliferation at 24 h remained relatively high at lower concentrations. At 9.375 µg/mL, proliferation was 94.8% ± 2.94, decreasing slightly to 92.39% ± 3.48 at 18.75 µg/mL and maintaining 95.9% ± 4.72 at 37.5 µg/mL. However, as the concentration increased, cytotoxicity became more pronounced, with cell proliferation dropping to 82.09% ± 10.26 at 150 µg/mL and a steep decline to 27.37% ± 6.28 at 300 µg/mL (Figure 4).

At 48 h, a similar pattern emerged, with high proliferation at lower concentrations (99.77% ± 10.29 at 9.375 µg/mL and 87.09% ± 9.46 at 18.75 µg/mL), while higher concentrations resulted in more significant cytotoxicity. At 150 µg/mL, cell proliferation was reduced to 62.76% ± 14.87, and, at 300 µg/mL, only 17.19% ± 6.05 of the cells remained viable (Figure 4). After 72 h, cytotoxic effects were more pronounced, with proliferation reduced to 45.29% ± 4.07 at 150 µg/mL and only 9.32% ± 3.87 at 300 µg/mL (Figure 4). The IC_50_ values for the infusion decreased over time, reflecting an increase in cytotoxicity: 236.2 ± 6.25 µg/mL at 24 h, 178.7 ± 9.26 µg/mL at 48 h, and 143.8 ± 6.38 µg/mL at 72 h.

For the HE, the effects were more pronounced across all concentrations and time points. At 24 h, proliferation at the lowest concentration (9.375 µg/mL) was 91.78% ± 8.65, and, at 18.75 µg/mL, it dropped to 85.89% ± 6.77. At 37.5 µg/mL, proliferation was 92.09% ± 6.71, but at 150 µg/mL and 300 µg/mL, proliferation fell sharply to 58.67% ± 9.99 and 22.07% ± 8.54, respectively (Figure 4). After 48 h, the cytotoxicity of the HE increased significantly, with 81.91% ± 10.51 cell viability at 9.375 µg/mL, 72.06% ± 8.75 at 18.75 µg/mL, and 64.11% ± 6.30 at 75 µg/mL. At higher concentrations, 150 µg/mL reduced proliferation to 40.18% ± 4.28, and, at 300 µg/mL, only 16.25% ± 5.64 of the cells remained viable (Figure 4). At 72 h, cytotoxicity was even more severe, with 90.80% ± 21.71 cell viability at 9.375 µg/mL but only 8.40% ± 2.58 remaining at 300 µg/mL (Figure 4). The IC_50_ values for the hydroethanolic extract showed a steep decline over time, reflecting its potent cytotoxic effects: 183.1 ± 9.31 µg/mL at 24 h, 93.11 ± 14.90 µg/mL at 48 h, and 80.34 ± 9.65 µg/mL at 72 h.

When comparing the two extracts (Figure 4), it becomes evident that the hydroethanolic extract (HE) is significantly more cytotoxic than the infusion (INF). The HE’s IC_50_ values are lower across all time points, indicating that it reduces cell viability more effectively at lower concentrations. For example, at 72 h, the IC_50_ for the HE is 80.34 ± 9.65 µg/mL, while for the INF, it is 143.8 ± 6.38 µg/mL, showing that the HE becomes much more toxic over time. Additionally, at the highest concentration of 300 µg/mL, the HE causes almost complete cell death by 72 h (8.40% ± 2.58 viability), whereas the INF, though still toxic, retains slightly more viable cells (9.32% ± 3.87 at 72 h). When comparing the effects of *Mentha aquatica* INF and HE on N27 dopaminergic cells with their effects on normal human dermal fibroblasts (NHDFs), clear differences in sensitivity and cytotoxicity become evident. Both cell types show dose- and time-dependent responses to the extracts, but the neural cells (N27) exhibit much greater vulnerability, especially to the HE.

In N27 cells, the HE demonstrates significantly lower IC_50_ values across all time points, indicating stronger cytotoxic effects. For example, at 72 h, the IC_50_ for HE is 80.34 ± 9.65 µg/mL, while for INF, it is 143.8 ± 6.38 µg/mL. This indicates that the HE exerts more severe cytotoxicity at lower concentrations. At 300 µg/mL, HE nearly wipes out the N27 cells, with only 8.40% ± 2.58 cell viability remaining at 72 h, compared to 9.32% ± 3.87 for the INF.

The fact that both extracts are significantly more toxic to N27 cells reflects the heightened sensitivity of neural cells to oxidative stress and mitochondrial dysfunction, which are likely induced by the bioactive compounds in the HE. Additional studies would help clarify the underlying molecular mechanisms of this toxicity and confirm whether these effects are directly attributable to specific compounds in the extracts. This further investigation would allow for a more robust validation of the hypothesis and provide greater insight into the safety and therapeutic potential of the extracts in sensitive cellular models like neurons. When we compare these results with the NHDF cells, the cytotoxic effects of both extracts are less pronounced. For NHDF cells, the IC_50_ values are higher, indicating that these cells are more resistant to the toxic effects of both the INF and HE. NHDF cells exposed to the HE for 72 h show a higher IC50 and greater viability compared to N27 cells, indicating that dermal fibroblasts are more resilient to oxidative stress and apoptotic triggers. Despite some toxicity, NHDF cells maintain higher proliferation rates, even at high concentrations of HE and INF, whereas dopaminergic neurons (N27 cells) are more sensitive to damage. This difference stems from the higher metabolic activity of neural cells, which rely heavily on mitochondrial function for ATP production [139]. Disruptions to mitochondrial health in these cells can amplify oxidative stress and ROS production, leading to damage [140]. In contrast, NHDF cells, with their metabolic flexibility and stronger antioxidant defenses, are better equipped to handle oxidative stress [141,142]. These findings highlight the need to account for cell-specific responses when developing therapies targeting oxidative-stress-related conditions.

## 4. Conclusions

In conclusion, *Mentha aquatica* demonstrates significant potential as a natural therapeutic agent, particularly in managing diabetes and oxidative stress. Through detailed chemical analysis using HPLC-ESI-MSn-DAD, 57 bioactive compounds were identified, including hydroxycinnamic acids, flavanones, hydroxy flavanones, methoxy flavone, and isoflavonoids. These compounds are known to contribute to various pharmacological properties, particularly antioxidant and enzyme inhibitory activities. The study revealed that both the infusion (INF) and hydroethanolic extract (HE) possess comparable polyphenol content, with similar antioxidant effects against DPPH and nitric oxide radicals. However, the INF showed superior superoxide radical scavenging capacity, which is crucial for neutralizing oxidative stress, a key factor in diabetic complications. This enhanced activity may be due to the distinct composition of compounds extracted by water in the infusion process, which favors the isolation of more potent hydrophilic antioxidants. In terms of enzyme inhibition, which is directly relevant for diabetes management, the INF displayed significantly better *α*-glucosidase inhibitory activity than the HE. The inhibition of *α*-glucosidase is an essential mechanism in controlling postprandial hyperglycemia, making INF a more effective candidate for managing blood glucose levels. The INF also outperformed the HE in preventing hemoglobin oxidation and lipid peroxidation, both of which are critical markers of oxidative damage and potential contributors to chronic diseases such as diabetes.

Furthermore, the cytotoxicity assays conducted on NHDF cells and N27 dopaminergic neurons indicated that the INF is less toxic compared to the HE, especially at higher concentrations and prolonged exposure times. While the HE demonstrated stronger cytotoxic effects, particularly in neural cells, the INF exhibited a safer profile, maintaining higher cell viability. This suggests that the infusion might be better suited for long-term use or as part of a daily regimen in functional foods or herbal therapies aimed at oxidative stress reduction and diabetes management.

*Mentha aquatica*, especially in its infusion form, emerges as a promising natural agent with hypoglycemic, antioxidant, and cytoprotective properties. Its ability to inhibit key enzymes involved in carbohydrate metabolism, combined with its relatively low cytotoxicity, positions it as a viable candidate for further development in therapeutic applications, particularly in the context of diabetes and oxidative damage mitigation. Further research should explore the clinical potential of *Mentha aquatica* extracts, particularly the INF, to confirm its efficacy in vivo for managing diabetes and related oxidative stress disorders. Studies assessing the long-term effects of *Mentha aquatica* extracts on glycemic control, oxidative markers, and inflammation could provide valuable insights. Additionally, isolating and characterizing individual bioactive compounds from the infusion might help identify those with the strongest effects, paving the way for developing targeted therapeutic agents. Investigating optimal extraction conditions, formulation techniques, and delivery methods could also enhance bioavailability and maximize therapeutic efficacy. The influence of environmental factors on the phytochemical composition of *Mentha aquatica* should be further examined to support sustainable cultivation practices that preserve or enhance its bioactive potential.

## Figures and Tables

**Table 1:** Calibration curves of external standards (concentrations range of 1.5–100 µg/mL) used in the quantification of phenolic compounds of *Mentha aquatica* extracts.

Phenolic Compound	Calibration Curve Equation		R^2^
Syringic acid	y = 9077x – 8779.4	Also used to quantify salvianolic acid B, B/E, and similar compounds	0.999
Rosmarinic acid	y = 3869.8x − 12610	Used to quantify galloyl derivatives and *p*-hydroxybenzoic acid derivatives	0.999
*p*-hydroxybenzoic acid (4-hydroxybenzoic acid)	y = 24.800x – 4376.7	Used to quantify naringenin derivatives	0.999
Hesperidin	y = 16.884x – 22.233	Used to quantify luteolin and apigenin derivatives	0.999
Luteolin	y = 2726.8x − 4670	Used to quantify luteolin derivatives	0.999
Quinic acid	y = 25.540x + 124.292	Also used to quantify ferulic acid derivatives	0.999
Caffeic acid	y = 74.093x + 53.692	Used to quantify 4-CQA and sinapoyl hexoside	0.999
Chlorogenic acid	y = 47.383x – 12.104	Used to quantify kaempferol derivatives	0.999
Kaempferol 3-*O*-glucoside	y = 13.085x + 13.444	Used to quantify ferulic derivatives	0.999
Ferulic acid	y = 21.172x – 9478.7	Used to quantify p-coumaroyl hexoside	0.999
*trans*-cinnamic acid (*m*-coumaric acid)	y = 17.721x + 12.1678	Also used to quantify salvianolic acid B and B/E and similar	0.999

**Table 2:** Yield extraction (%) and TPC (total phenolic contents) (mg GAE/g DW extract) of infusion (INF) and hydroethanolic extracts (HE) of *Mentha aquatica*.

Sample Extract	Yield (%)	TPC (mg GAE/g DW) *
INF	16.210 ± 6.690	170.290 ± 8.505
HE	12.113 ± 0.021	177.078 ± 4.842

* GAE (gallic acid equivalent); DW (dry weight).

**Table 3:** Retention time (Rt), mass spectral data, and identification of the chemical compounds found in *Mentha aquatica* infusion (INF) and hydroethanolic extract (HE) by HPLC-ESI-MS-MS.

N°	RT	Molecular Formula	[M + H]+ (*m*/*z*)	MS/MS Fragments (*m*/*z*)	Compound Name	Nature of Compound
*Mentha aquatica* infusion (INF)						
1	8.34	C_27_H_30_O_15_	594.1572	287.0544, 449.1071, 85.0287	Kaempferol 3-*O*-rutinoside	Flavonoid glycoside
2	8.65	C_9_H_6_O_3_	162.0310	63.024, 89.039, 95.049, 135.044	5-hydroxycoumarin	Coumarin
3	9.44	C_8_H_10_O_7_	218.0393	95.049, 51.024, 105.045, 77.039	6-*O*-acetylascorbic acid	Ascorbic acid Derivative
4	9.87	C_12_H_21_NO_2_	211.1564	55.055, 91.055, 79.055, 141.070	Elaeokanine C	Alkaloid
5	10.09	C_10_H_8_O_3_	176.0465	65.039, 149.023, 121.029, 111.044	7-methoxycoumarin	Coumarin
6	10.53	C_14_H_24_O_7_	304.1512	68.9976, 129.0181, 185.0804, 255.4873	Creoside I	Acyclic monoterpenoids
7	10.54	C_9_H_12_O_4_	184.0728	68.998, 129.018	Antiarol	Phenol
8	10.79	C_17_H_26_O_4_	294.1822	57.0705, 73.0289, 221.1167, 133.101	Vanillyl nonanoate	Aromatic ester
9	11.18	C_18_H_28_O_4_	308.1977	57.071, 221.117, 15.039, 107.049	5-*O*-methyl embelin	Benzoquinone Derivative
10	12.55	C_18_H_30_O	262.2287	67.0548, 81.0703, 95.0858, 105.0702	Farnesylacetone	Sesquiterpene ketone
*Mentha aquatica* hydroethanolic extract (HE)						
11	7.96	C_27_H_32_O_15_	595.1657	289.0702, 85.0287, 195.0286, 435.1274	Neoeriocitrin	Flavanones
12	7.98	C_15_H_12_O_6_	287.0552	153.018, 89.0389, 163.0387, 135.0439	Eriodictyol	Flavanones
13	8.00	C_21_H_22_O_10_	433.1131	195.0287, 85.0288, 245.0437, 135.044	Naringenin-8-*O*-glucoside	Flavanones
14	8.34	C_27_H_30_O_15_	593.1501	287.0544, 449.1071, 85.0288	Nictoflorin	Dihydroxyflavanone
15	8.36	C_15_H_10_O_6_	285.0394	153.018, 89.0389, 68.9976, 135.0439	Kaempferol	Tetrahydroxyflavone
16	8.46	C_21_H_18_O_12_	461.0714	287.0544, 153.0179	Scutellarin	Glycosyloxyflavone
17	8.65	C_9_H_6_O_3_	161.0238	63.0235, 89.0389, 117.0335	4-hydroxy coumarin	Coumarins
18	8.69	C_27_H_30_O_14_	577.1551	271.0594, 433.1123	Kaempferitrin	Glycosyloxyflavone
19	9.68	C_19_H_20_O_8_	375.1074	213.039, 216.0261, 198.0157, 139.0025	Hyperinone	Polyketides
20	10.09	C_10_H_8_O_3_	175.0393	65.0391, 149.0232, 121.0286, 111.0444	7-methoxycoumarin	Coumarins
21	10.17	C_17_H_16_O_6_	315.0865	183.0286, 133.0647, 161.0595, 168.0048	Persicogenin	Flavanones
22	10.20	C_18_H_18_O_7_	345.0968	213.0389, 216.0259, 198.0156, 55.0185	Amorphaquinone	Isoflavonoid
23	10.75	C_18_H_16_O_7_	343.0813	315.0494, 169.0129, 154.9973, 201.0025	Ayanin	Trimethoxyflavone
24	10.78	C_17_H_26_O_4_	293.1748	57.0705, 73.0289, 221.11164, 101.0236	Nordihydrocapsiate	Phenols
25	10.84	C_19_H_20_O_7_	359.1125	227.0545, 197.0078, 169.0129, 215.0182	Elaeocyanidin	Leucoanthocyanidins
26	11.09	C_20_H_20_O_8_	387.1074	359.0755, 169.0128, 341.0647, 197.0076	Combretol	Pentamethoxyflavone
27	11.42	C_19_H_18_O_7_	357.0979	329.065, 169.0129, 311.0546, 197.0079	Retusin	Tetramethoxyflavone

**Table 4:** Quantification of phenolic compounds (µg/g of plant extract) identified in plant treated with methanol 50% + formic acid 1% (A) and infusion (INF) and hydroethanolic extracts (HE) of *Mentha aquatica* by HPLC-DAD-ESI-MSn.

N°	Compound Identification	Nature of Compound	Formula	λmax (nm)	Molecular Ion [M − H] (*m*/*z*)	Fragments MS/MS (*m*/*z*)	Quantification (µg/g)		
							Mentha Treated (A)	INF	HE
1	Syringic acid	Methoxybenzoic acid	C_9_H_10_O_5_	276	197	182, 153	2032.25 ± 29.99	n.q	n.q
2	3-*O*-caffeoylquinic acid	Hydroxycinnamic acid ester	C_16_H_18_O_9_	326	353	193, 181	n.q	n.q	n.q
3	Caffeic acid hexoside (1)	Hydroxycinnamic acid glycoside	C_15_H_18_O_9_	238, 328	341	179, 163	n.q	n.q	n.q
4	Caffeic acid hexoside (2)		C_15_H_18_O_9_	238, 328	341	179, 163	n.q	n.q	n.q
5	Caffeic acid hexoside (3)		C_15_H_18_O_9_	238, 328	341	179, 163	n.q	n.q	n.q
6	Icariside b	Flavonoid glycoside	C_19_H_32_O_8_	-	506	507, 147	n.q	n.q	n.q
7	Medioresinol	Lignan	C_21_H_23_O_7_	-	387	207	n.q	n.q	n.q
8	Eriodictyol-7-*O*-rutinoside	Flavanone glycoside	C_27_H_32_O_15_	285	595	287	n.q	n.q	n.q
9	Luteolin-7-*O*- rutinoside		C_27_H_30_O_15_	240, 288, 328	593	285	n.q	n.q	n.q
10	Luteolin-7-*O*-glucoside		C_21_H_20_O_11_	234, 328	447	285	n.q	n.q	n.q
11	Luteolin-7-*O*-glucuronide		C_21_H_18_O_12_	256, 348	461	287	n.q	n.q	n.q
12	Naringenin-7-*O*-rutinoside		C_27_H_32_O_14_	280, 330	579	435, 419, 273	n.q	n.q	n.q
13	Apigenin-7-*O*-rutinoside		C_27_H_30_O_14_	268, 334	577	269, 225, 201	1776.62 ± 13.48	n.q	n.q
14	Salvianolic acid B/E isomer	Polyphenolic acid	C_36_H_30_O_16_	238, 330	717	555, 519, 475, 357, 295	n.q	n.q	n.q
15	Hesperetin-7-*O*-rutinoside	Flavanone glycoside	C_28_H_34_O_15_	285, 332	609	286, 177, 151	852.88 ± 0.71	n.q	n.q
16	Rosmarinic acid	Caffeic acid ester	C_18_H_16_O_8_	238, 328	359	197, 179, 161	n.q	n.q	n.q
17	Dimethyl caffeic acid	Hydroxycinnamic acid derivative	C_11_H_12_O_4_	323, 294	207	135	n.q	n.q	3100.73 ± 13.78
18	Dimethyl caffeic acid derivative		C_11_H_12_O_4_	-	-	-	n.q	n.q	4744.83 ± 34.00
19	Dimethyl caffeic acid hexose		C_17_H_22_O_9_	280	371	209, 191, 163	n.q	2620.23 ± 18.12	nq
20	Ferloyl derivative		C_10_H_10_O_4_	310, 325	193–195	178, 149, 134	n.q	485.20 ± 10.39	nq
21	Ferulic acid derivative 1		C_10_H_10_O_4_	-	-	-	n.q	n.q	70,297.35 ± 392.71
22	Ferulic acid derivative 2		C_10_H_10_O_4_	-	-	-	n.q	n.q	98,324.02 ± 783.94
23	Ferulic acid derivative 3		C_10_H_10_O_4_	-	-	-	n.q	n.q	7880.64 ± 255.60
24	Feruloyl hexose		C_16_H_20_O_9_	320	355	193,160	535.87 ± 4.14	n.q	n.q
25	Feruloyol derivative		C_10_H_10_O_4_	-	-	-	n.q	12,473.91 ± 165.60	n.q
26	Galloyl derivative	Gallic acid derivative	C_7_H_6_O_5_	-	-	-	n.q	35,273.99 ± 45.58	n.q
27	*p*-hydroxybenzoic acid derivative	Phenolic acid derivative	C_7_H_6_O_3_	-	-	-	n.q	24,588.37 ± 103.65	n.q
28	Quercetin	Flavonol	C_15_H_10_O_7_		301	227	7119.73 ± 4.33	n.q	n.q
29	Quercetin derivative	Flavonol derivative	C_15_H_10_O_7_	-	-	-	9086.20 ± 22.60	n.q	n.q
30	Quinic acid	Cyclohexanecarboxylic acid	C_7_H_12_O_6_	330	191	171, 173	n.q	21,151.90 ± 83.26	n.q
Ʃ							21,403.55	96,593.60	184,347.57

(A): methanol 50% + formic acid 1%. n.q: detected but not quantified.

**Table 5:** In vitro biological activities of the infusion (INF) and hydroethanolic extracts (HE) of *Mentha aquatica*. The results are presented as mean IC_50_ (µg/mL) ± standard deviation (n = 3).

Sample	DPPH	SO	NO	A-GLUC	Hb_ox	LP_ox
INF	20.353 ± 0.563	96.484 ± 3.251	11.073 ± 1.237	20.917 ± 1.632	52.638 ± 0.168	267.662 ± 15.827
HE	38.875 ± 0.693 *	281.417 ± 21.019	12.73 ± 0.123 *	354.179 ± 19.275 *	128.711 ± 13.274 *	490.611 ± 25.305 *
ASC	6.720 ± 0.123	142.4228 ± 6.356	112.567 ± 8.407	-	-	-
ACA	-	-	-	17.0175 ± 0.977		-
QUE	-	-	-	-	369.701 ± 18.185	2.607 ± 0.064

DPPH: 2,2-diphenyl-1-picrylhydrazyl (DPPH^•^) assay, NO: nitric oxide (^•^NO) assay, SO: superoxide radical scavenging (O_2_^•−^) assay, Hb_ox: hemoglobin oxidation assay, LP_ox: lipid peroxidation assay, A-GLUC: *α*-glucosidase activity, ASC: ascorbic acid, ACA: acarbose, QUE: quercetin. * indicates significant differences between extracts (*p* < 0.05) using Student’s *t*-test analysis.

## Data Availability

The data presented in this study are available on request from the corresponding author due to due to privacy/ethical restrictions or institutional policies.

## Supplementary Materials

The following are available online at https://www.mdpi.com/article/10.3390/antiox13121512/s1. Table S1: Results of HPLC-ESI-MS-MS of *Mentha aquatica* infusion and hydroethanolic extracts.
